# Effects of different acupuncture treatment methods on post-stroke cognitive impairment: study protocol for a multicenter randomized controlled trial

**DOI:** 10.1186/s13063-020-04959-y

**Published:** 2021-01-06

**Authors:** Kai-Qi Su, Su-Tong Liu, Jie-Ying Li, Rui-Qing Li, Hui-Li Feng, Yang Xue, Xiao-Dong Feng

**Affiliations:** 1 Henan University of Chinese Medicine, No. 156 East Jinshui Road, Zhengzhou, 450046 China; 2 Rehabilitation Center, The First Affiliated Hospital of Henan University of Chinese Medicine, No. 19 Renmin Road, Zhengzhou, 450000 China

**Keywords:** Acupuncture, Cognitive impairment, Stroke, Clinical trial

## Abstract

**Background:**

Cognitive impairment is a common dysfunction after stroke that seriously affects the overall recovery of patients. Cognitive rehabilitation training is currently the main treatment to improve cognitive function, but its curative effect is limited. Acupuncture is a core component of traditional Chinese medicine (TCM), and some previous clinical studies have shown that it might be effective in treating post-stroke cognitive impairment (PSCI), but further evidence from large-sample studies is needed. The overall objective of this trial is to obtain further data to develop an optimized acupuncture treatment for PSCI by comparing the effects of different acupuncture treatment methods on cognitive function in PSCI patients.

**Methods/design:**

In this multicenter, prospective, randomized controlled trial, 206 eligible stroke inpatients who meet the trial criteria will be randomly assigned to 2 groups: an electroacupuncture (EA) plus needle retention (NR) group and an EA group. Both groups of patients will undergo the same routine cognitive rehabilitation treatments. All treatments will be given 5 times per week for 8 weeks. The primary outcomes will be assessed using the Mini-Mental State Examination (MMSE) and the Montreal Cognitive Assessment scale (MOCA). The secondary outcome will be measured by the Barthel Index (BI). All outcomes will be evaluated at baseline, week 4, week 8, and the third and sixth month after the end of treatment.

**Discussion:**

Our aim is to evaluate the effects of two different acupuncture treatment methods for treating PSCI patients. This study is expected to provide data to be used in developing an optimized acupuncture treatment method for PSCI treatment.

**Trial registration:**

Chinese Clinical Trial Registry ChiCTR1900027849. Registered on 30 November 2019, http://www.chictr.org.cn/showproj.aspx?proj=46316

## Background

Stroke is the leading cause of death and disability worldwide and has a rising incidence and a high disability rate, often accompanied by many types of dysfunction [1–3]. Post-stroke cognitive impairment (PSCI) is a series of syndromes that meet the diagnostic criteria for cognitive impairment within 6 months after the occurrence of stroke and is observed after approximately 66% of strokes [4]. Furthermore, one study reported that the incidence of PSCI can be as high as 69.8% within 3 months of stroke [5]. PSCI mainly manifests as disruptions in advanced brain functions such as learning, memory, executive ability, and visual space/structural ability, which seriously affect patient quality of life (QOL) and overall rehabilitation [6, 7].

Management of vascular risk factors (hypertension, hyperlipidemia, diabetes, smoking, new atrial fibrillation and other arrhythmias) is critical, and the use of antiplatelet drugs (aspirin, clopidogrel, etc.) and anticoagulants is recommended according to the stroke prevention guidelines [8, 9]. In addition, the use of angiotensin-converting enzyme inhibitors and diuretics can delay the progression of white matter changes in patients with stroke [10]. Donepezil, galantamine, and memantine have been reported to improve cognitive function, but there are adverse reactions and certain risks [11–13]. A Cochrane review analyzed the efficacy of nondrug interventions focusing on cognitive rehabilitation for PSCI, and the results showed that more research evidence is needed [14]. Hyperbaric oxygen therapy and Tai Chi may contribute to cognitive improvements, but have not yet been proven to be effective independent therapies [15, 16]. Although there are multiple treatments, there are still few effective treatments for PSCI.

Acupuncture is a core component of TCM, and it has a definite effect on PSCI [17–19]. However, there are shortcomings such as numerous acupuncture points, inconsistent treatment times, and unclear cumulative effects of acupuncture and needle retention. At present, no unified acupuncture treatment method has been released. In addition, research on the dose-effect relationship of acupuncture is increasing year by year. However, whether needle retention can improve the efficacy of conventional EA therapy in patients with PSCI remains unclear. Therefore, to study the clinical efficacy and safety of EA and NR for PSCI, we designed this multicenter, prospective, large-sample randomized controlled trial with a sufficient follow-up period. As a preliminary experiment, we aim to observe the effects of EA treatment on cognitive function by subjective and objective assessments and to obtain further data to develop an optimized acupuncture treatment for PSCI. The results will be helpful to demonstrate whether EA plus NR treatment is an effective and safe therapy for improving cognitive function in patients with PSCI.

## Trial objectives

1. To observe and compare the difference between the two treatment methods in improving cognitive impairment and activities of daily living in patients with PSCI.
2. To provide more evidence for developing an optimized acupuncture treatment method for PSCI treatment in the future.

## Methods/design

### Trial design

**Fig. 1:**
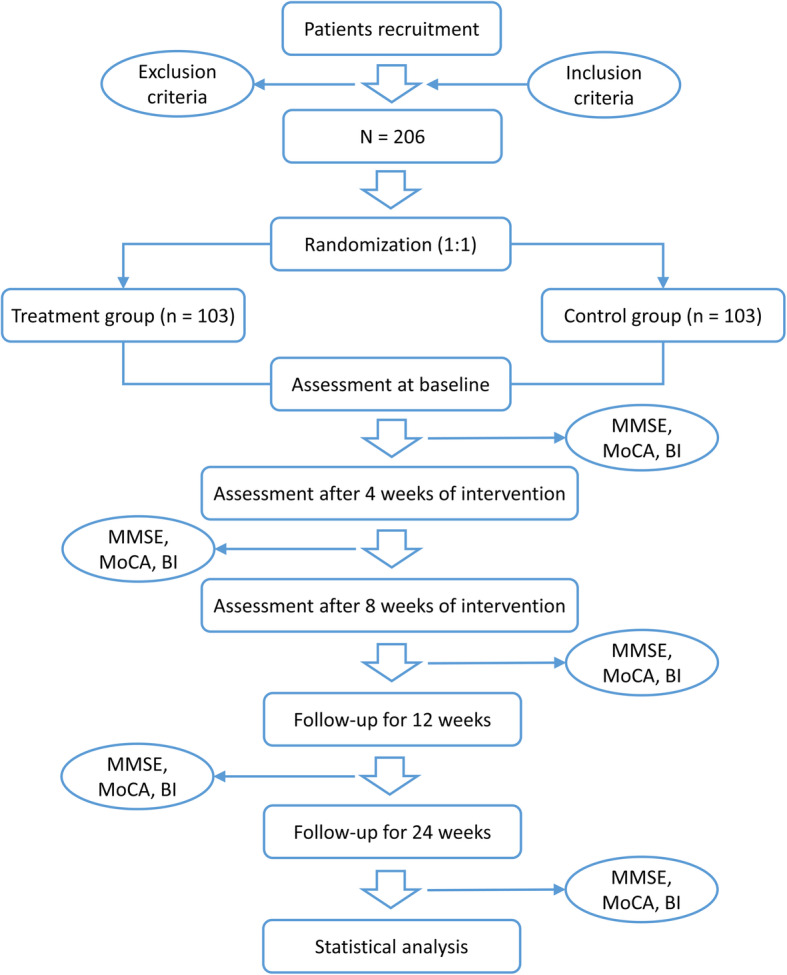
Flowchart of the trial

This is a multicenter, prospective, randomized controlled trial supported by the Henan Administration of Traditional Chinese Medicine. The trial will be jointly conducted by three centers in Zhengzhou, China: the First Affiliated Hospital of Henan University of Chinese Medicine, the Henan Province Hospital of TCM, and the Third Affiliated Hospital of Henan University of Chinese Medicine. A total of 206 patients who meet the predefined criteria will be randomly assigned to 2 groups, with one group receiving EA plus NR and routine rehabilitation treatment and the other group receiving EA plus routine rehabilitation treatment. After a 1-week washout period, patients will receive treatment 5 times a week over 8 weeks of observation. Participants will be assessed at 4 time points: baseline (0 weeks), the middle of the treatment (4 weeks after treatment starts), the end of the treatment (8 weeks after treatment starts), and follow-up (12 weeks and 24 weeks after treatment finishes). All participants will complete the MMSE, MOCA, and BI assessments. The study flow chart is shown in Fig. 1. The trial process chart is shown in Table 1.

**Table 1:** Timing of treatment assessments and data collection

	Study period					
	Enrolment	Baseline	Treatment phase		Follow-up phase	
Timepoint	− 1 week	0 week	4 weeks	8 weeks	12 weeks	24 weeks
Enrolment						
Eligibility screen	×					
Informed consent	×					
Medical history	×					
Merger disease	×					
Randomization		×				
Interventions						
Treatment group		×	×	×		
Control group		×	×	×		
Assessments						
MMSE		×	×	×	×	×
MOCA		×	×	×	×	×
BI		×	×	×	×	×
Safety evaluation		×		×		
Adverse events		×	×	×	×	×

### Ethics

This trial was approved by the ethics committee of the First Affiliated Hospital of Henan University of Chinese Medicine on 5 September, 2019 (reference number: 2019HL-102-01). All participants will sign an informed consent form, and to protect their privacy, their real names will not appear in related reports.

### Study setting

All participants in this trial will be recruited from the First Affiliated Hospital of Henan University of Chinese Medicine, the Henan Province Hospital of TCM, and the Third Affiliated Hospital of Henan University of Chinese Medicine. Interventions for all participants will be carried out in the hospitals where the participants are recruited. The First Affiliated Hospital of Henan University of Chinese Medicine will be responsible for this trial coordination and data management.

### Sample size

We will conduct this study using a randomized controlled trial design, and the main observed outcome will be improvements in patient cognitive function. Our previous small-sample (20 cases) clinical observation found that 2 weeks of EA plus routine cognitive rehabilitation treatment and EA plus NR and routine cognitive rehabilitation treatment can reduce scores on the MOCA scale by 3.17 ± 2.12 and 4.40 ± 2.59 points, respectively. With the significance test level set at 0.05 and the test power at 0.9, the sample size was calculated by using the following:

$$ N=2\times {\left[\left({z}_{\alpha /2}+{z}_{\beta}\right)\times \upsigma /\updelta \right]}^2 $$

*N* is the required sample size for each group, and the sample size of each group is equal. When *α* is 0.05 and *β* is 0.1, the normal distribution quantile table shows the following:

$$ {Z}_{\alpha /2}=1.96 $$

and

$$ {Z}_{\beta }=1.282. $$

*σ* and *δ* represent the larger standard deviation and the mean difference between two groups, which are 2.59 and 1.23, respectively. By substituting the above data into the formula, it was calculated that 93 patients will be needed in each group. Accounting for a 10% dropout rate, the final estimated sample size is approximately 103 patients in each group (206 in total).

### Inclusion criteria

Participants meeting the following criteria will be included:

1. Male or female participants aged 30–75
2. Participants who meet the diagnostic criteria of stroke by computed tomography (CT) or magnetic resonance imaging (MRI)
3. Participants with cognitive impairment identified with the MMSE scale and MOCA assessment scale
4. Patients with cognitive impairment that occurred after stroke, and not for other reasons
5. Patients with a disease course between 1 month to 1 year who did not use any cognitive enhancing drugs before enrollment
6. Patients with clear consciousness and stable vital signs
7. Patients who voluntarily agree with the investigation and sign a written informed consent form for the clinical trial

### Exclusion criteria

Participants who report any of the following conditions will be excluded:

1. Patients with non-stroke diseases that can cause cognitive impairment such as intracranial space occupying lesions, brain trauma, and aneurysms
2. Patients with diseases that seriously affect cognitive tests, such as abnormal audiovisual functions and mental disorders
3. Patients with a significant history of cognitive decline or dementia prior to this stroke onset
4. Patients with other serious diseases such as immune diseases, endocrine diseases, and abnormal liver and kidney functions
5. Pregnant or lactating women
6. Patients participating in other clinical trials that could affect the evaluation of this study’s results

### Elimination criteria

1. Patients who do not meet the criteria for a diagnosis of PSCI and are mistakenly included
2. Patients with poor compliance or self-withdrawal, or patients who are judged by the investigator as unsuitable to continue the trial because of serious adverse complications

### Recruitment

The main participants in this trial will be recruited simultaneously through the outpatient and inpatient systems of the three centers, and we will also recruit online through websites and hospital-based WeChat ads of the three centers. Posters, pictures, and videos related to the study will be produced to help participants understand the purpose of this study, while explaining the advantages and disadvantages of the treatment and what they need to do to participate in this study. Based on the inclusion/exclusion criteria, we will make a preliminary judgment and screening of the possibility of inclusion of participants who are interested in participating in the study, and determine whether the subject is included according to the results of the examination. Participants who meet the inclusion criteria will be informed of the study details, and subjects or their legal guardians will be required to sign an informed consent form before treatment begins. The recruitment of participants began on 1 December 2019 and is expected to end in June 2021, or it may be completed early if a sufficient number of patients are recruited.

### Randomization

All included patients will be randomly divided into two groups at a ratio of 1:1: the EA plus NR group and the EA group. A specialized statistician who is not a researcher in this study will be entrusted to use randomization software to generate random-number sequences for randomization. Meanwhile, the allocation should be hidden. Strips identifying participants by grouping information will be hidden in sealed opaque envelopes with sequential numbers. The envelopes will not be opened until informed consent is obtained. Each clinical research center will select cases strictly according to the inclusion and exclusion criteria, will determine the eligible patients who can be included, and then will obtain random numbers and grouping information by phone until the total number of patients (206) is reached.

### Implementation

A specialized statistician who is not a researcher in this study will generate the allocation sequence. Relevant independent researchers in each center will enroll participants and assign participants to different interventions.

### Blinding

Due to the limitations of this protocol and the nature of acupuncture treatment, we were unable to adopt a double-blind study design. To improve the quality of this study as much as possible, an outcome-assessor-blinded trial procedure will be implemented. With the exception of the evaluation process, the evaluator will not contact any participants. In addition, to prevent nonblind participants or researchers from biasing the outcomes, this study will only involve individuals with no conflicts of interest or preconceived positions. In the process of data management and statistical analysis, we will invite independent professionals to handle data management, and the statistical analysis of all data will be performed by professional statisticians who are not the principal researchers.

### Intervention

Participants in the two groups will receive different treatments 5 times a week for 8 weeks in separate compartments. All acupuncturists will be physicians with at least 3 years of experience in acupuncture practice. Meanwhile, each participant will receive the same routine cognitive rehabilitation treatments from therapists who will be blinded to the treatment allocation. The interventions in the two groups are as follows:

#### EA plus NR group

In addition to routine cognitive rehabilitation treatments, participants in this group will receive EA treatment. The regular acupuncture method will be applied at Baihui (GV20) and Shenting (GV24). With the patient in the supine position, a 75% alcohol cotton swab will be used to routinely disinfect the two acupuncture points, and Huaying stainless steel acupuncture needles (0.25 mm × 25 mm) will be used, keeping the angle between the needle tip and the scalp at 30°, and the needle tip will be moved backwards along the anterior-posterior midline, and the needle will be inserted 0.5 cun. After insertion, manipulations will be applied for the “Deqi” sensation. The EA therapeutic apparatus (G6805-2A, Shanghai Huayi Medical Instrument Co., Ltd., China) will be connected to the needles at the two acupuncture points with 15 min of continuous waves and 15 min of dense waves at a frequency of 2.5 HZ. After a total of 30 min of stimulation, the EA therapeutic apparatus will be removed. Then, the needles will remain at the acupuncture points for 1 h, and manipulations will be performed every 30 min until the needles are removed.

#### EA group

In addition to routine cognitive rehabilitation treatments, the intervention of this group will be the same as that in the EA plus NR group except that the needles will be directly removed after 30 min of electrical stimulation. There will be no NR process.

#### Routine cognitive rehabilitation treatments

According to the actual situation of all participants in the two groups, we will follow the theory of neuropsychology and provide each participant with the same cognitive rehabilitation treatment through a combination of artificial training and computer training that will include classification exercises, rule (response) suppression exercises, plan analysis exercises, reasoning comprehension exercises, working memory exercises, and comprehensive ability exercises.

### Outcome measures

All participants will be evaluated at these time points: baseline, week 4, week 8, and follow-up week 12 and week 24 after all treatments are completed. All treatment evaluations will be conducted by researchers who will be blinded to the treatment allocation.

#### Primary outcomes

##### Mini-mental state examination (MMSE)

The MMSE scale is the most widely used cognitive function assessment questionnaire and has the advantages of simplicity, time savings, convenient operation, and relevance for a wide range of people [20]. It consists of 30 questions including orientation, memory, calculation, recall, and language. Each answer is worth 1 point for a total of 30 points. The assessment criteria for cognitive impairment vary with the patient’s educational level: illiterate or semi-illiterate ≤ 17 points, primary school ≤ 20 points, and middle school ≤ 24 points. The lower the MMSE scale score is, the worse the cognitive function is considered.

##### Montreal cognitive assessment scale (MOCA)

The MOCA is currently one of the generally acknowledged cognitive impairment screening scales [21], which mainly includes items such as attention and concentration, executive function, memory, speech, visual structure, abstract thinking, computing power, and orientation. Compared with the MMSE, the MOCA has better sensitivity and specificity for patients with mild cognitive impairment. The total possible score is 30 points. If the subject has been educated for 12 years or less, 1 point is added, and a score of < 26 points is considered to indicate cognitive impairment.

#### Secondary outcome

##### Barthel Index (BI)

BI is a questionnaire for assessing activities daily living [22]. It mainly includes the ability to control urination, eating, wearing, walking, bathing, and other daily life activities. Each item has a score of 0, 5, 10, or 15 points based on the degree of disruption, and the total possible score is 100 points. A higher score indicates a worse living ability. BI classification is as follows: heavy dependence (score 0–40), moderate dependence (41–60), light dependence (61–99), and without dependence (100).

### Safety evaluation and adverse events

The safety evaluation mainly refers to the evaluation of safety indicators and adverse events. The safety indicators will be tested once before and after the treatment and will include a general physical examination (blood pressure, breathing, pulse), routine blood tests, routine urine tests, routine stool tests, electrocardiograms, and liver and kidney function tests. Adverse events in this study will be defined as any discomfort, symptoms, or diseases that occurred during this acupuncture clinical trial, such as fainting, allergies, or pain. If someone faints, researchers will immediately remove needles, then keep the patient in a supine position in a ventilated place, and give him/her warm water or sugar water to let him/her fully rest until the body recovers. If allergies or pain occurs, needles will be immediately withdrawn, and the patient will be treated symptomatically. All the above detailed information regarding safety evaluations will be reported in detail in case report forms (CRFs), and the impact of all adverse events will be analyzed at the end of the study. Participants who experience adverse events will be compensated accordingly.

### Data management

All information of participants will be truthfully, completely, accurately, and timely recorded in CRFs, and codes and initials will be used to replace the information of participants. Special personnel will be asked to manage the relevant data, and each participant’s personal information will be kept strictly confidential. Without the explicit permission of the person in charge of the research group, data will not be shared with third parties other than data recorders and data administrators. At the end of the trial, study participants will submit the CRFs on time, and then, the quality control team members will check the completeness and accuracy of the CRFs.

### Quality control

To guarantee the research quality of this trial, we will invite a qualified clinical trial expert from the Clinical Research Center of the First Affiliated Hospital of Henan University of Chinese Medicine to monitor this study, and detailed regulations for each link of data collection and management will be formulated to clarify the responsibilities of researchers. If any problems in the project are identified, the center will decide to change the research plan after approval by the application ethics committee. A reasonable and feasible data standard operating procedure (SOP) will be established to ensure quality at each stage of data collection and management. Data management will be carried out by relevant personnel who have been trained and operate strictly in accordance with the SOP. Meanwhile, a quality control team will be established to conduct quality control and inspections on a regular basis. The qualified clinical trial expert will monitor the work of the clinical trial center at least once a month.

### Statistical analysis

Statistical analysis of all data will be performed using SPSS 22.0 software by specialized statisticians who are not the principal researchers. Classified variables will be analyzed using Pearson’s *χ*^2^ test or Fisher’s exact test, and continuous variables will be evaluated using Student’s *t* test or an appropriate nonparametric method. The measurement data from different treatment groups will be statistically described by the mean ± standard deviation. When comparing within groups or between groups, a normality test and homogeneity of variance test will be performed before data analysis of each group. If the data satisfy the normal distribution and homogeneity of variance, then the *t* test will be used for comparison between two groups, and the LSD or SNK method will be used for multiple comparisons. Conversely, the rank-sum test will be used for nonnormality or nonuniformity of variance. The significance level used for statistical analysis with 2-tailed testing will be set at 5%.

## Discussion

The effective improvement of cognitive impairment after stroke is particularly important to improve the rehabilitation and quality of life of patients after stroke, so it is necessary to take effective treatment measures to improve cognitive function. Acupuncture therapy has a history of more than 2000 years in China and has been widely used in Western countries in recent years. Our previous clinical research found that EA treatment at Baihui (GV20) and Shenting (GV24) along with NR on the head can effectively relieve cognitive impairment after stroke. However, the best acupuncture treatment mode and the cumulative effects of acupuncture still need further research and confirmation. Therefore, we propose a prospective, multicenter, large-sample randomized controlled trial to explore the cumulative effect of acupuncture and needle retention. This study is expected to provide a preliminary basis for the optimization of treatment methods for PSCI, to further develop a standardized, simple, effective, and easy-to-promote comprehensive TCM clinical rehabilitation treatment method.

Although most of the previous studies on acupuncture treatment of cognitive impairment used a combination of body acupuncture points and head acupoints [23–25], we will use only two acupuncture points on the head (GV20 and GV24) for treatment, which is based on the results of literature analysis and a continuation of previous research [26, 27]. In addition, unified acupuncture points may reduce variability and increase the accuracy and persuasiveness of this study. In recent years, the MMSE, MOCA, and BI scales have been used to assess various aspects of cognitive function, so we used these three items as outcome indicators to reduce the interference of subjective factors and simplify the assessment of cognitive impairment. In addition, the multicenter design of the study may introduce a bias, mainly by including differences between acupuncture operator techniques. Therefore, to eliminate the bias caused by operator inconsistency, we will conduct unified training for all the acupuncturists participating in this study and strictly regulate the acupuncture operation to maximize the elimination of this bias. However, although all results will be measured and recorded by independent researchers to minimize the risk of detection bias, as a limitation of this protocol, we cannot use double-blind procedures to conduct this study due to the nature of acupuncture treatment.

To achieve our clinical goals, we will strive to standardize every step of this study. We expect that this trial will provide strong evidence for acupuncture treatment of PSCI, with a view to incorporating or updating the corresponding TCM rehabilitation guidelines and further formulate a clinical rehabilitation treatment method suitable for grassroots promotion and application.

### Dissemination policy

After study completion, the researchers will submit the final data to the Henan Administration of Traditional Chinese Medicine in the form of a research report. The findings will be shared with healthcare professionals, the general public, and relevant organizations through the publication of manuscripts and conference presentations.

### Trial status

The recruitment of patients for this study began on 1 December 2019, and this study is ongoing. Protocol version number and date: V2.0, 1 September 2019. The recruitment of patients is expected to be completed in June 2021.

## Data Availability

Not applicable; no data have yet been generated.

## Abbreviations

BI: Barthel Index
CRF: Case report form
CT: Computed tomography
EA: Electroacupuncture
MMSE: Mini-Mental State Examination
MOCA: Montreal Cognitive Assessment scale
MRI: Magnetic resonance imaging
NR: Needle retention
PSCI: Post-stroke cognitive impairment
QOL: Quality of life
SOP: Standard operating procedure
TCM: Traditional Chinese medicine
